# Environmental Performances of Various CCU Options in the Framework of an Integrated Chemical Plant

**DOI:** 10.3390/membranes11110815

**Published:** 2021-10-26

**Authors:** Olivier Mirgaux, Hélène Anselmi, Fabrice Patisson

**Affiliations:** Institut Jean Lamour, Labex Damas, Université de Lorraine, 54011 Nancy, France; helene.anselmi@solvay.com (H.A.); fabrice.patisson@univ-lorraine.fr (F.P.)

**Keywords:** LCA, process modelling, carbon capture and utilization (CCU), adsorption, absorption, membranes

## Abstract

Several carbon capture processes are investigated to separate a part of the CO_2_ contained in the flue gas of a coal-fired power plant located in a chemical integrated plant, with the objective of using it as a raw material in a production process. The expected results are to reduce the impact on global warming potential (GWP) and to increase the productivity of the plant. The study is based on the modelling of the combination of systems in the plant using a process simulation software and using life cycle assessment to evaluate both technical feasibility and environmental aspects. Models for the power plant, the production processes, amine chemical absorption, membrane separation and adsorption on activated coal are developed and validated against industrial and literature data. The life cycle inventory is obtained from the mass and energy balances given by the systems model. A first set of calculations is launched with a high purity requirement for the CO_2_ stream (95%) recycled into the process. Those calculations show a 12% increase in productivity for the chemical process considered, but result in no significant gain in terms of GWP. Conversely, scenarios with a lower CO_2_ purity (40%) show a drop around 9% of the impacts on GWP using membrane separation and activated coal adsorption, while keeping the other impacts at about the same level.

## 1. Introduction

Among the numerous environmental issues the chemical industry sector has to face, global warming will be one of the most challenging in coming next decades. Keeping productivity, product quality and production costs stable while reducing greenhouse gases (GHG) emissions is a major topic to tackle the global warming issue and anticipate the evolution of the carbon market in the next decades.

Many carbon capture (CC) technologies are now entering into their mature phase, offering new opportunities for cleaner production at the industrial scale. Nonetheless capturing CO_2_ raises the question of the fate of the captured CO_2_. Storage, for example geological storage (CCS), is of course a possibility, but it is often associated with animated debates in the scientific community and in the civil society [1]. Moreover, when stored, CO_2_ becomes another burden to be looked at. A more satisfying option, when it is possible, is to consider the captured CO_2_ as a new raw material and to find ways to use it (Carbon Capture and Utilization—CCU) [2].

In the framework of the present study, we consider the particular case of an integrated chemical plant, which is responsible for large CO_2_ emissions, mainly because of the presence of a coal-fired power plant onsite which paradoxically requires CO_2_ as raw material in the chemical process itself. With such a configuration, CCU appears to be particularly well adapted to both reduce CO_2_ emissions and valorize the captured CO_2_ onsite. As we worked on an existing plant, post-combustion options, which are easier to implement in brownfield projects, were considered. Chemical absorption with solvents is a mature technology [3], which was first implemented at industrial scale in Denmark on a power station with monoethanolamine (MEA) [4]. Other options using solutions of demixing amines were studied in [5,6,7,8] and showed a better energetic efficiency of the process. Other solvent types were also investigated [9,10,11]. Membrane processes were deeply investigated in [12,13,14] and a recent paper [15] showed that they could be more advantageous than absorption processes in terms of environmental impacts. Adsorption on activated coal, zeolites or grafted nanofibrous adsorbents may be operated with various configurations [16,17,18,19]. Both membranes and adsorption processes compete with chemical absorption in terms of performances. Among these processes we decided to consider one process of each category to produce a CO_2_ stream from the flue gas of the power plant that could be eligible for direct utilization in the chemical process: chemical absorption with MEA, which is the most mature process [2], polymer membrane separation, and temperature swing adsorption (TSA) on activated coal.

Our work was then divided into three main tasks. First, a complete model of the plant was built using the process simulator Aspen Plus and validated against industrial data [20]. This model includes the different part of the chemical process itself and the coal-fired power plant as well. Models of the selected CC technologies were then developed using Aspen Plus and Aspen adsorption [21] and incorporated in a global Aspen Plus flowsheet representative of the whole plant. Such an approach ensures that all the mass and energy balances are respected and that the CC facilities considered are well designed to fit all the requirements and constraints of the considered plant. In the last part of the study, which is the heart of the present paper, we used the results of the Aspen calculations as a basis to build the life cycle inventory (LCI) of the different scenarios and we proceeded to a comparative life cycle assessment (LCA) of those scenarios. This hybrid methodology, which associates systems modelling and LCA was already adopted several times [22,23,24,25] in other contexts. It allows the design and the comparison of processes in terms of environmental impacts with very limited use of generic commercial data bases, which may not be relevant in such specific configurations. To our knowledge, it is the first time this methodology is applied to compare chemical absorption, polymer membrane separation and temperature swing adsorption in the framework of their potential integration on an industrial plant for CCU.

## 2. Materials and Methods

### 2.1. Plant Overview

**Figure 1:**
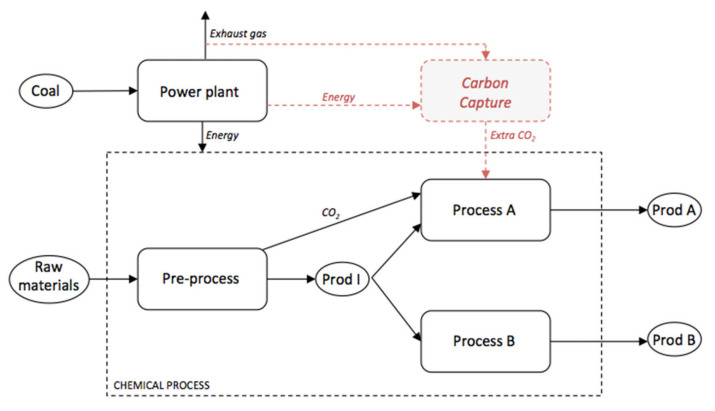
Schematic representation of the considered plant.

The chemical plant considered is composed of several processes that can be schematically gathered in four main parts: the coaled-fired power plant, a pre-process that produces an intermediate product (Prod I), and two main processes (A and B) producing two different products (Prod A and Prod B respectively), as shown in Figure 1.

The production of Prod A, which is a high-quality grade product for pharmaceutical applications, requires CO_2_. This CO_2_ is provided by the pre-process (co-product) and is a limiting reactant in the chemical reactions involved in process A, which means that amount of Prod A produced is bounded by the amount of CO_2_ collected at the pre-process. Prod B is of lower quality and does not require CO_2_ for its production. The actual plant (without CC) produces 3.15 tons of Prod B for 1 ton of Prod A. The whole process is supplied in energy by the coal-fired power plant onsite.

In this paper, we consider the possible addition of a CC unit: a limited amount of CO_2_ from the exhaust gas of the power plant would be recovered to supply process A with extra CO_2_ and increase Prod A yield (marked in red in Figure 1). At the same time, a reduction in GWP impacts could be expected. The CC facilities would be supplied in energy (electricity and steam) by the power plant.

To be eligible for plant integration, the CC facilities should have a minimal CO_2_ production capacity of 1 ton per hour with a minimal CO_2_ recovery of 75%. In addition, the minimal CO_2_ purity should be 95 mol%, according to specific requirements of Prod A. Given those requirements, around 13 tons of exhaust gas from the power plant would be processed to produce 1 tCO_2_/h. The detail of the chemical process and its modelling are not described here for confidentiality reasons.

### 2.2. Power Plant

The coal-fired power plant was modelled on Aspen Plus. The model is schematically divided into two sub models—the boiler and the steam cycle—which are connected.

Coal is considered a ‘non-conventional solid’ described by its chemical composition, diameter (0 to 50 mm), humidity (10%) and lower heating value (28,100 kJ/kg). In the model, the coal is dried before entering a ‘R-Yield’ reactor where it is decomposed into chemical elements. Combustion is then performed into a ‘R-Gibbs’ reactor. The Redlich Kwong-Soave thermodynamic state equation is used to describe the gas phase into the boiler. The hot combustion gas is sent to heat exchangers where its energy is transferred to the steam cycle. The steam cycle is modelled by a seven-stage (two high pressure, twp medium pressure and three low pressure) Hirn cycle with overheated steam delivering 20 MWhe. Isentropic efficiencies are respectively 90, 92 and 85% for high, medium and low-pressure turbines, respectively. Electromechanical efficiency is set to 95%. The vapour mass fraction at the end of the cycle is 0.78 at 30 °C and 50 mbar. At each stage water is condensed and collected before starting a new cycle.

The exhaust gas is cleaned from dust through filters before being sent to the de-NOx and de-SOx processes. As NOx and SOx are considered to be very harmful to the considered CC facilities, we decided to enhance the de-NOx and de-SOx processes of the actual plant to be able to reach a negligible concentration of NOx and SOx in the flue gas. De-NOx is performed through Selective Non-Catalytic Reduction (SNCR) with ammonia solution (25 wt-%). The flow rate of the ammonia solution was increased by 18% in comparison to the reference process. De-SOx on the plant is achieved using dry slaked lime produced onsite (intermediate product in the pre-process). This process was completed by an additional treatment in a column with a soda solution (20 wt-%). Beforehand the gas was cooled down to 50 °C in a cooler. This additional treatment requires 4.75 GJ of steam, 1120 kg of demineralized water and 280 kg of NaOH to produce 1 ton of CO_2_ per hour; those values are given in the case of membrane separation and slightly change depending on the calculated performances of the considered process.

The main results of the simulation of the power plant are reported and compared to onsite-measured data in Table 1.

The calculated results are in good agreement with measurements. As mentioned above, SOx and NOx concentrations are negligible in our model because of the enhanced de-NOx and de-SOx treatments considered. The extra ammonia required for the enhanced de-NOx treatment explains the difference of ammonia consumption between the model and the plant. The noticeable difference between the calculated and measured ash production should be attributed to the variability of the coal composition on the plant, which changes with the supply, whereas it was fixed in the model. The concentration of CO_2_ of the flue gas is relatively low, around 8.5 wt-%. This model was connected to the global flowsheet of the plant on Aspen Plus, and automatically adapts its energy production to supply the various facilities of the plant.

### 2.3. CC Technologies Considered

- Membrane separation

The membrane separation process was modelled using MEMSIC, a third-party module [26], which was integrated in the Aspen Plus model. Different configurations were tested and a two-stage arrangement of two different polymer membranes was selected to achieve the specifications [21].

**Figure 2:**
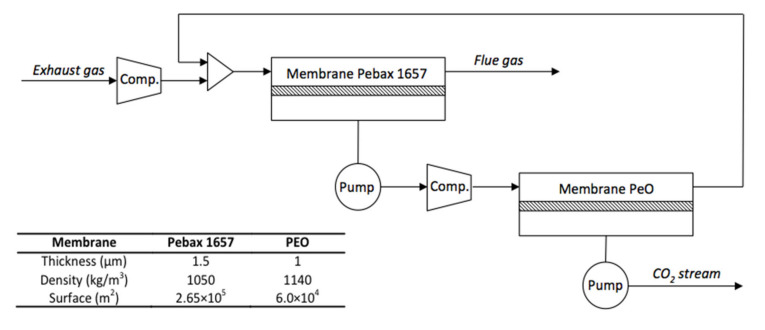
Schematic representation of the selected membrane process.

The polymer membranes were chosen on the basis of their performances, their well-known physicochemical properties, and their availability: polyethylene oxide (PEO), which is highly selective, and Pebax 1657, which is highly permeable to CO_2_ [27]. These membranes are associated to a compressor and a vacuum pump as shown in Figure 2. The pressure of the gas fed at the membrane entrance is 1.5 bar and the pressure of permeate is 0.15 bar. The properties of the two membranes, reported in the figure, were calculated from the model to reach the desired performances.

- Chemical absorption with MEA

The chemical absorption with MEA was modelled on Aspen Plus as well. A rate-based kinetic approach was employed and heat and mass transfers inside each column were considered. Details of the model can be found in [21] and main characteristics of the process are reported in Table 2.

- Temperature swing adsorption

The third CO_2_ capture process, TSA on activated carbon, was modelled [21] with Aspen Adsorption, through a dynamic approach, using cycles for simulating the successive adsorption and desorption steps. For a given column, the cycle consists of four steps: adsorption, regeneration (the bed is heated indirectly by steam circulating in pipes inside the bed), a hot gas purge with CO_2_ to avoid CO_2_ dilution and N_2_ cooling. The process is made of three columns to allow a continuous operation. The main characteristics of the columns and the process operating parameters are indicated in Table 3.

### 2.4. Life Cycle Assessment

The goal of the present study is to evaluate from an environmental point of view the whole plant in its standard operating conditions (without CC, reference scenario) and to assess the potential benefit of the integration of the different CC options onsite. A LCA following the ISO14040 and 14044 standards [28,29] was carried out in both cases using the commercial software GABI 8. The life cycle inventory was mainly established on the basis of the computer simulations conducted on Aspen Plus software described above. These data were completed for standard processes by the commercial database ecoinvent 3.5 integrated to GABI 8.

The functional unit is “production of 1 ton of product A and 3.15 tons of product B”, which is representative of the standard operation of the plant. Calculations showed that with the implementation of CC facilities, the rate of production of product A would be increased of 12% without any change regarding the production of Prod B. This means that with CO2 capture and valorisation, for 1 ton of Prod A, 2.8 tons of Prod B is produced. To stick to the functional unit, an additional production of 0.35 ton of Prod B onsite is considered (boundaries extension).

Four scenarios were considered: reference case without CCU, plant with chemical absorption with MEA, plant with membrane separation and plant with adsorption on activated carbon (TSA).

The other LCA assumptions are as follows. The construction and end of life of the infrastructure of the chemical process and of the power plant are not considered. The coal comes from Russia and is transported to the plant by train and trucks. The collected ashes are buried. The life duration of the membranes is 5 years. The degraded membranes are not recycled but directly incinerated. The activated carbon comes from Russia and has to be changed every 5 years. The degraded MEA from purges is collected and incinerated.

The impact assessment was performed with the method ReCiPe v1.08 midpoint indicators. Considering the nature of the processes at study and the associated emissions, we focused on the following categories of impacts: global warming potential (GWP100), eutrophication potential (EP), freshwater eutrophication (FEP), terrestrial acidification (TAP), particulate matter formation potential (PMFP), photochemical oxidant formation potential (POFP), ozone depletion potential (ODP), human toxicity potential (HTP), freshwater ecotoxicity potential (FETP) and terrestrial ecotoxicity potential (TETP).

## 3. Results

### 3.1. LCI

The inventory was calculated on the basis of the results of the simulations conducted with our models. The main flows of materials and energy calculated are listed below, for the different units of the plant.

- Power plant

The principal results of the inventory (per functional unit, FU) for the power plant in the reference scenario are given in Table 4 for the functional unit. Depending on the scenario considered, the production of electricity is of course subject to variations, which are accounted for in the following.

- Production processes

The main results for the inventory (per functional unit, FU) of the production processes under the reference scenario are reported in Table 5.

- Carbon capture facilities

The flows of materials and energy associated to the use phase of the different CC facilities are given in Table 6.

### 3.2. Impact Assessment

**Figure 3:**
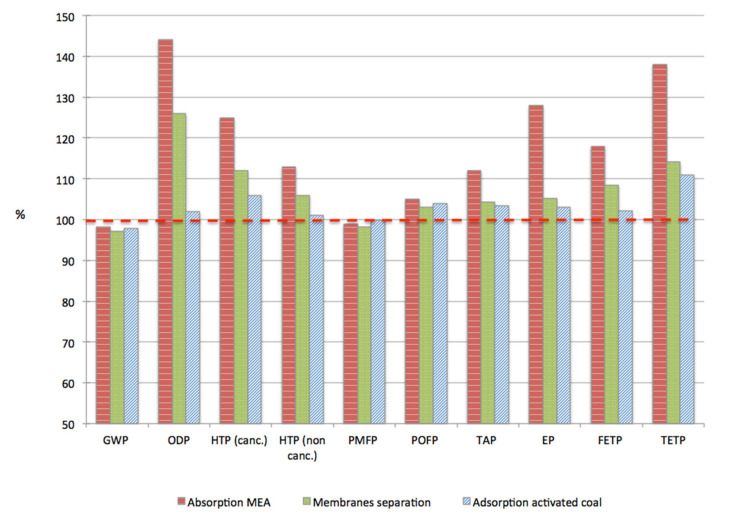
Comparison between the different CC scenarios and the reference case (set to 100%) for the following impacts: global warming potential (GWP100), eutrophication potential (EP), freshwater eutrophication (FEP), terrestrial acidification (TAP), particulate matter formation potential (PMFP), photochemical oxidant formation potential (POFP), ozone depletion potential (ODP), human toxicity potential (HTP), freshwater ecotoxicity potential (FETP), terrestrial eco-toxicity potential (TETP).

Figure 3 compares the four scenarios considered in terms of their impacts. In this graph, the reference scenario corresponding to the actual plant without CCU is set to 100% and the other scenarios are reported in terms of their relative environmental impacts.

Globally, one observes that for all the selected categories of impacts, the results are very close to the reference case or worse; no significant improvement is observed in any category.

**Figure 4:**
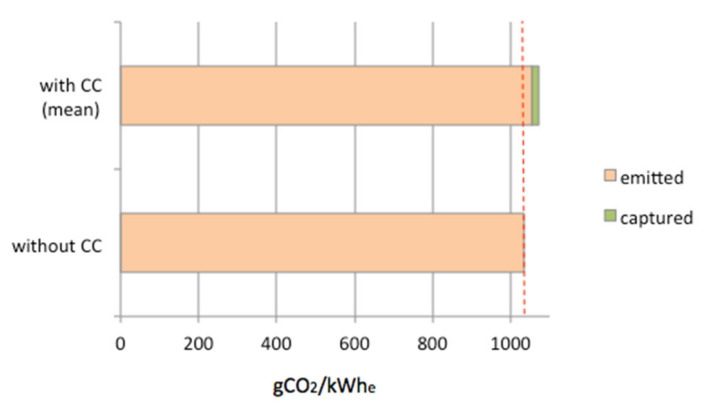
CO_2_ emitted by the coal-fired power plant.

Concerning GWP, which is the main concern of the present study, the potential impacts for the four scenarios are in a nutshell. Chemical absorption, membranes and adsorption on activated coal reach 98.2, 97.2 and 97.8% of the reference case, respectively. This negligible and non-significant gain results from two factors: the energy required to run CC facilities, which erases the benefice of the CO_2_ recycling, and the relatively low amount of CO_2_ captured and reused onsite. Indeed, the CC facilities were designed to capture 1000 kg/h of CO_2_, which hardly represents 2% of the standard emissions of the coal-fired power plant. At the same time, the production of Prod A is increased by the CO_2_ recycling by around 12%, which means that a substantial improvement in term of GWP could be expected, after rescaling to the functional unit. However, it appears that the extra energy required to operate the CC facilities strongly mitigates the results. This is clearly illustrated on Figure 4, which represents the amount of CO_2_ generated by the power plant to produce 1 kWh_e_ with (mean value) and without carbon capture. This graph shows that the implementation of CC facilities results in net CO_2_ emissions.

Similar conclusions can be derived regarding the particulate matter formation potential (PMFP) category, which is directly linked to the emissions associated to the production of energy with the coal-fired power plant. The category photochemical oxidant formation potential (POFP) is also strongly linked to the energy production through the SOx emissions resulting from the combustion of coal, but one has to keep in mind that the absolute value of the indicator for this category is relatively weak due to the strong de-SOx purification process operated.

Absorption with MEA exhibits significantly higher results than the other processes regarding the eutrophication potential (EP), freshwater eutrophication potential (FETP), terrestrial eutrophication potential (TETP), ozone depletion potential (ODP) and terrestrial acidification potential (TAP). These results are in agreement with the literature [30]. ODP category is strongly affected by the ethylene oxide associated to the production of MEA, and by the degradation of MEA during CO_2_ separation, which represents 22% and 78% of the impacts associated to the absorption process in this category, respectively. The degradation of MEA also strongly affects EP, TETP and TAP categories. The end-of-life treatment of the solvent rather impacts FETP and EP.

The membrane technology is slightly better than MEA absorption in most categories of impacts, which is consistent with the results published in [31]. The ozone depletion potential (ODP) is strongly impacted by emissions of chlorofluorocarbides and hydrofluorocarbides during the incineration of the membranes and to a lesser extent by the emissions associated de production of the membranes. These emissions represent 52% and 28% of the impacts associated to the membrane process in this category, respectively. The production of the membranes is also responsible for emissions of hydrogen fluoride, which affects the human toxicity potential category.

Adsorption on activated coal, which appears to be the most attractive alternative, presents impacts relatively close to the reference case in most categories. The differences mainly come from the extra energy required to operate the facility. To our knowledge no dedicated literature investigated the environmental assessment of this technology.

Those results are globally disappointing and especially in terms of global warming potential. The integration of the CC facilities improves the production of Prod A but offers no benefice in terms of CO_2_ emissions and more or less significantly increases the impacts in all the other categories for the given functional unit.

### 3.3. Parametrical Study

The previous calculations were conducted strictly meeting the performances criteria of the CC facilities previously presented. As those criteria led to unsatisfying results regarding the goal of the study, we decided to conduct a parametrical study to try to find a compromise. For each process, we identified one key parameter, which could be modified without compromising the separation process. The principal results are presented below.

- Absorption with MEA

A significant part of the impacts associated to the MEA process comes from MEA and its degradation products released during CO_2_ separation. Direct releases of MEA originate from the purge implemented in the process to maintain a constant quality of the solvent. Consequently, reducing the purge rate has a positive impact regarding the MEA releases and the global MEA consumption of the process, but at the same time it decreases the efficiency of the process because of the accumulation of degraded products in the solvent. We conducted calculations for a reduction of 15% in the purge rate and correspondingly the consumption of extra MEA was reduced by 5%, the CO_2_ recovery and the CO_2_ purity fell to 73% and 85%, respectively. Those values are still satisfying but unfortunately in terms environmental impacts, the benefice does not exceed 5% of reduction for ozone depletion potential and freshwater ecotoxicity potential and is not significant for the other categories, especially regarding GWP.

- Membranes

The impacts associated to the membrane process mainly come from the energy consumption during separation and from the materials (polymers) of the membranes. We investigated thinner membranes switching from 1.5 μm to 0.07 μm for the Pebax 1657 and from 1 to 0.03 μm for the PEO, which resulted in significant material savings. Reducing the membranes thickness maintain a good efficiency of the process but implies a drop of 11% in the CO_2_ purity.

Impacts are globally unchanged except for ozone depletion and human toxicity (canc.), which are reduced by about 5%.

- Adsorption on activated coal

For this process, the impacts are mainly associated to the energy required to heat the bed and to a lesser extent to the activated coal itself. We tried to reduce the amount of activated coal from 10%, which implied a drop of 13% in the CO_2_ recovery. As expected, this modification hardly reduces the environmental impacts.

- Membranes and activated coal with 40% CO_2_ purity

The previous results clearly showed that a significant decrease in the different categories of impacts, and especially in GWP, could not be reached with a simple tuning of processes operating parameters. Nevertheless, we further thought about the minimal performances criteria of the separation processes established at the beginning of the study. The minimal CO_2_ production capacity and the minimal CO_2_ recovery rate are imposed by technical constraints that cannot be modified. However, it appeared that the CO_2_ purity, which was first set to 95% to ensure a standard high purity, could be reduced without damage to the process A. Process A actually requires a very clean CO_2_ to ensure the high purity of Prod A required by its market. But this purity is disconnected from the concentration of CO_2_ in the feed gas. On the actual plant, process A is fed with a CO_2_ stream, which is purified from unwanted components (dust, SOx and NOx) but which is highly diluted: the CO_2_ molar fraction does not exceed 40%. Consequently, as the tail gas collected on the power plant is highly purified prior to CO_2_ separation, it could be eligible for process A with a molar fraction considerably lower than the 95% considered previously. Two additional scenarios, with membrane process and chemical adsorption on activated coal, were then considered with a CO_2_ molar fraction of 40%. Chemical absorption with MEA, which appeared to be the less promising process in terms of environmental impacts, was not considered in the following.

Reducing the purity requirements to 40% considerably simplify the membrane separation process, as only one stage of Pebax 1657, without recycling loop, is enough to reach this concentration. The total surface of membranes and the energy required to operate the process are drastically reduced (by about 70% and 60%, respectively). The separation process with activated coal is also deeply impacted: a smaller quantity of coal is required (66% less), which leads to a strong cut in the energy required to heat the bed and run the process (60% less). The main characteristics of the new processes are reported in Table 7.

**Figure 5:**
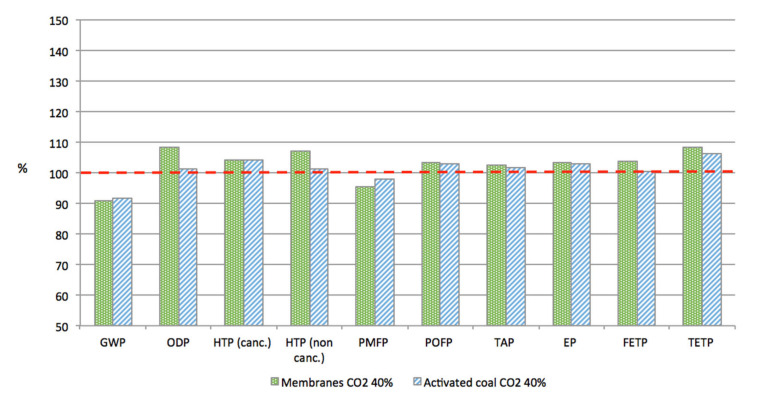
Comparison of the alternative scenarios with the reference case (set to 100%), CO_2_ at 40%. Impacts: global warming potential (GWP100), eutrophication potential (EP), freshwater eutrophication (FEP), terrestrial acidification (TAP), particulate matter formation potential (PMFP), photochemical oxidant formation potential (POFP), ozone depletion potential (ODP), human toxicity potential (HTP), freshwater ecotoxicity potential (FETP), terrestrial eco-toxicity potential (TETP).

The results for these two alternatives are reported in Figure 5. This graph clearly shows a significant drop of the results in all the considered categories of impacts, compared to the previous calculations with a CO_2_ purity of 95%. With a CO_2_ purity of 40%, both membranes and activated coal options lie within the same range of impacts as the reference case. Impacts on GWP are now significantly lower than the reference case, with a 9% reduction for the membranes and an 8% reduction for the activated coal. This drop is mainly attributed to the saving in the energy required during CO_2_ separation. Consequently, a significant decrease in the particulate matter formation potential impacts, which are directly linked to the energy consumption, is observed as well. For all the other categories, the impacts remain higher than for the reference case, though they are drastically reduced in comparison to scenarios with a CO_2_ purity of 95%.

For all the categories considered, except for GWP and particulate matter formation potential, the membranes have higher impacts than the activated coal.

## 4. Discussion

The present study aimed at reducing the impacts on GWP associated to the production of two commercial products (A and B) of a chemical plant, while increasing the production of the most valued product (Prod A) through a partial capture of CO_2_ from a coal-fired power plant and its reuse as a raw material for the production of Prod A. The calculations conducted with a high purity CO_2_ (95%) showed a significant improvement, around 12%, of the yield of Prod A but the expected decrease of the potential impacts on GWP was unfortunately not achieved. This was explained by an extra requirement of energy to operate the different facilities considered for CO_2_ separation, which almost totally erased the benefice of the gain of productivity of Prod A and of the capture of a portion of the CO_2_ released into the atmosphere. Furthermore, the impacts in all the other categories (except particulate matter formation potential) were significantly increased, which led us to conclude that despite the higher yield of Prod A, which may have an economic interest to the long term, the investigated options were not satisfying from an environmental point of view.

The parametrical study conducted on a limited set of parameters of the three CC processes considered showed that no significant improvements could be expected without reconsidering the minimal requirements imposed at the beginning of the study. Consequently, a new set of calculations were conducted for the most promising options—membrane and activated coal—with a CO_2_ purity of 40%, which could be eligible for process A. With no surprise, the characteristics of the new separation processes are highly simplified (only one stage without recycling for membrane and a smaller column with less coal for adsorption) and led to a drastic saving in the energy consumed for CO_2_ separation. With this new configuration, which presents the same advantage as previously in terms of Product A yield, both membranes and activated coal led to a significant drop in terms of GWP, around 9% compared to the reference case without CCU. This result is quite satisfying as only 2% of the CO_2_ from the power plant is captured. In addition, all the results for the other categories of impacts are only 5% to 18% higher than the reference case, which is quite less than in the case with a CO_2_ purity of 95%.

## 5. Conclusions

Although there are small differences in the different categories of impacts for membrane separation and adsorption on activated coal, it is not possible to choose one of these processes above the other one. The differences in the results are very weak and the uncertainties both in our modelling and in the impact assessment method do not allow ranking these processes in terms of environmental impacts. Both options are promising and only technical or economic considerations could lead to a choice.

One important conclusion of this study, which is independent of the application, is that CCU should not be taken as an ideal solution to reduce the environmental load of processes. CC technologies have proven their efficiency for drastically reducing the impact on GWP of standard power plants, while increasing the impacts in other categories such as acidification and human toxicity, as highlighted in [30]. However, when the CC is done for CCU, the results are highly dependent on the local context. In our study, despite an actual gain in terms of impacts on GWP (minus 9% for the case with a 40% CO_2_ purity), the very low amount of the processed flue gas (only 2% of the CO_2_ is captured) drastically limited the potential of environmental gains.

## Figures and Tables

**Table 1:** Results of simulations for 1 MWhe.

Compounds	Model	Plant Data
Coal (kg)	1059.8	1058.9
Natural gas (kg)	23.3	21.8
Ammonia (kg)	25.3	21.4
Demineralized water (m^3^)	7426.1	7426.5
Process water (m^3^)	2443.1	2445.0
Condensate (m^3^)	787.0	803.5
Electricity produced (MWhe)	1	1
Steam produced (GJ)	24.5	25.0
Ash (kg)	109.7	125.6
Flue gas temperature (°C)	50	50
Flue gas pressure (atm)	1	1
Flue gas composition (%m)		
CO_2_	8.5	8.5
N_2_	74.1	74
H_2_O	7.1	7
O_2_	10.3	10.5
SOx	0	0.015
NOx	0	0.007

**Table 2:** Main characteristics of the chemical absorption with MEA.

Column Properties		
	Absorber	Stripper
Column type	FLEXIPAC, 250Y, Koch, Metal	FLEXIPAC, 250Y, Koch, Metal
Volume (m^3^)	58.9	45.2
Head losses (bar)	0.1	0.2
Number of stages *	20	20
**Operating Parameters**		
Stripper pressure (bar)	2.0	
MEA conc. (%mass)	30.0	
Solvent flowrate (m^3^/h)	38.0	
Extra MEA adding (kg/h)	1.41	
MEA purge (kg/t_CO2_)	0.38	

* including two stages of scrubbing.

**Table 3:** Column properties and operating parameters for the TSA process.

Column Properties			
Particle diameter	3.8 mm	Specific heat capacity	880 J kg^–1^ K^–1^
Particle porosity	0.46	Density	1138 kg m^–3^
Particle tortuosity	2.2	BET surface	1053 m^2^ g^–1^
Columns height	1.2 m	Columns diameter	4.8 m
**Operating Parameters**			
**Adsorption**			
Stop criteria	x_CO2_ ≥ 2 mol % in the output gas		
**Regeneration**			
Steam temperature	180 °C	Bed temperature	150 °C
**Purge with Hot CO_2_**			
Duration	120 s	CO_2_ temperature	150 °C
**Cooling with N_2_**			
Stop criteria	Bed temp. ≤ 30 °C	Gas temperature	30 °C

**Table 4:** Calculated inventory (main results) for the power plant (reference scenario).

Inputs/FU				Outputs/FU			
Materials (t)		Power		Materials (t)		Energy	
Coal	1.68	Electricity from the grid-French mix (GWh_e_)	0.05	CO	6.28 × 10^−3^	Elec. (GWh_e_)	1.41 × 10^−3^
				CO_2_	4.54		
				NOx	4.82 × 10^−3^		
Demineralizedwater	11.83			SOx	7.65 × 10^−3^		
				HCl	1.62 × 10^−4^		
				Lead	1.70 × 10^−6^		
				Mercury	6.84 × 10^−8^		
Cooling water	3.89	Electricity self-consumed (GWh_e_)	0.34	Chromium	3.91 × 10^−7^	Steam (GJ_th_)	32
				Arsenic	3.65 × 10^−6^		
				Nickel	3.08 × 10^−7^		
Ammonia	0.005			PM10	9.08 × 10^−4^		
				Dust	9.57 × 10^−4^		
				Ash	0.17		

**Table 5:** Calculated inventory (main results) for the production processes.

Input (/FU)		Energy (/FU)		Output (/FU)	
Pre-process					
Brine (t)	6.88	Elec. (GWh_e_)	6.9 × 10^−4^	Prod I (t)	4.42
Limestone (t)	7.04	Steam (GJ)	1.56 × 10^1^	CO_2_ (t)	9.11 × 10^−1^
Coke (t)	1.89			CO (t)	1.16 × 10^−1^
Ammonia (t)	5.31 × 10^−3^			Ca^2+^ (t)	1.70
Process water (t)	1.13 × 10^1^			Cl^−^ (t)	4.18
Anthracite (GJ)	1.19 × 10^1^			Na^+^ (t)	7.73 × 10^−1^
				Ammonia (t)	4.77 × 10^−3^
				SO_4_^2−^ (t)	3.09 × 10^−2^
Process A					
Prod I (t)	9.11 × 10^−1^	Elec. (GWh_e_)	8.46 × 10^−5^	Prod A (t)	1
CO_2_ (t)	6.49 × 10^−1^	Steam (GJ)	1.36	CO (t)	2.89 × 10^−2^
Process water (t)	1.09				
Process B					
Prod I (t)	3.52	Elec. (GWh_e_)	2.07 × 10^−4^	Prod B (t)	3.1
		Steam (GJ)	1.36 × 10^1^		

**Table 6:** Main material and energy flows associated to the use phase (CO_2_ separation) of the CC facilities (per functional unit).

Membrane Separation		Chemical Absorption		Temp. Swing Adsorption	
PEO (kg)	5.21 × 10^−4^	MEA in loop (t)	2.29	Activated coal (t)	4.21 × 10^−1^
Pebax 1657 (kg)	9.93 × 10^−5^	MEA losses (t)	6.0 × 10^−2^	Process water (t)	0.36
Process water (t)	3.50 × 10^−1^	MEA purges (t)	2.0 × 10^−2^	Energy (GJ)	1.95 × 10^−1^
Energy (GJ)	1.74 × 10^−1^	Energy (GJ)	2.3 × 10^−1^		

**Table 7:** Characteristics of membrane and adsorption processes with CO_2_ at 40%.

Membrane	
Surface of membrane (m^2^)	1.28 × 10^5^
CO_2_ production capacity (kg/h)	1011
CO_2_ recovery (%)	75.2
Energy requirements (GJ/FU)	6.85 × 10^−2^
**Adsorption**	
Activated coal (kg)	1.12 × 10^4^
CO_2_ production capacity (kg/h)	1008
CO_2_ recovery (%)	75.0
Energy requirements (GJ/FU)	7.68 × 10^−2^

## Data Availability

Not applicable.
